# How good can our beamlines be?

**DOI:** 10.1107/S0907444912034658

**Published:** 2012-09-18

**Authors:** Dorothee Liebschner, Miroslawa Dauter, Gerold Rosenbaum, Zbigniew Dauter

**Affiliations:** a Synchrotron Radiation Research Section, MCL, National Cancer Institute, Argonne National Laboratory, Argonne, IL 60439, USA; b SAIC-Frederick Inc., Basic Research Program, Argonne National Laboratory, Argonne, IL 60439, USA; c Department of Biochemistry, University of Georgia and Structural Biology Center, Argonne National Laboratory, Argonne, IL 60439, USA

**Keywords:** diffraction data precision, signal-to-noise ratio, measurement uncertainty, beamline performance

## Abstract

The accuracy of X-ray diffraction data depends on the properties of the crystalline sample and on the performance of the data-collection facility (synchrotron beamline elements, goniostat, detector *etc.*). However, it is difficult to evaluate the level of performance of the experimental setup from the quality of data sets collected in rotation mode, as various crystal properties such as mosaicity, non-uniformity and radiation damage affect the measured intensities. A multiple-image experiment, in which several analogous diffraction frames are recorded consecutively at the same crystal orientation, allows minimization of the influence of the sample properties. A series of 100 diffraction images of a thaumatin crystal were measured on the SBC beamline 19BM at the APS (Argonne National Laboratory). The obtained data were analyzed in the context of the performance of the data-collection facility. An objective way to estimate the uncertainties of individual reflections was achieved by analyzing the behavior of reflection intensities in the series of analogous diffraction images. The multiple-image experiment is found to be a simple and adequate method to decompose the random errors from the systematic errors in the data, which helps in judging the performance of a data-collection facility. In particular, displaying the intensity as a function of the frame number allows evaluation of the stability of the beam, the beamline elements and the detector with minimal influence of the crystal properties. Such an experiment permits evaluation of the highest possible data quality potentially achievable at the particular beamline.

## Abstract 2

A repetitive measurement of the same diffraction image allows to judge the performance of a data collection facility.

## 1. Introduction

The accuracy of measured diffraction data depends on the properties of the crystal and on the performance of the experimental setup. In small-molecule crystallography, in which crystals are characterized by well formed lattices, low mosaicity and high resistance to radiation damage, the data may reach very high accuracy when the intensities are measured with four-circle diffractometers and scintillation counters, leading to models refined with reliability factors of lower than 1% (Eichhorn *et al.*, 1991 ▶). In macromolecular crystallography, obtaining a very high accuracy of diffraction data is more difficult. Indeed, protein crystals are easily radiation-damaged, which is especially acute at contemporary very bright synchrotron X-ray beams even if the crystals are maintained at cryotemperatures. Protein crystals, especially when cryocooled, display a substantial level of mosaicity and are often non-uniform throughout their whole volume. It is therefore difficult to judge the level of performance of the data-collection facility (*i.e.* all synchrotron beamline elements, goniostat, detector, shutter *etc.*) from the quality of complete data sets collected in the rotation mode, in which the crystalline sample changes its orientation with respect to the X-ray beam. Comparison of reflection intensities measured multiple times in a series of diffraction images recorded using the same crystal orientation may lead to a more objective assessment of the quality than comparison of the intensities of symmetry-equivalent reflections within a complete or even a highly redundant data set. In the former situation the only crystal variable is the effect of radiation damage, which is expected to be smoothly monotonic, whereas in the latter case many additional effects come into play such as varying crystal diffracting volume, absorption and inhomogeneous radiation damage arising from rotation of the sample and the variation in scattering of the mounting loop and vitrified solvent while the crystal rotates. The quantitative effect of errors in the data sets collected in the rotation mode was recently investigated by Diederichs (2010 ▶), who analyzed in this context a series of data sets from the JCSG (Joint Center for Structural Genomics) archive. It was concluded that the accuracy of the strongest low-resolution reflections is mainly limited by the systematic errors resulting from the experimental setup, not by the influence of the crystal. The highest asymptotic value of the signal-to-noise ratio, (*I*/σ)^asymptotic^, was proposed as a useful indicator of the data quality. This ratio is inversely related to the *R*_merge_ value for the most intense low-resolution reflections. The analyzed JCSG data sets were characterized by (*I*/σ)^asymptotic^ values in the range of about 20–30, corresponding to an *R*_merge_ of 3–4%.

We performed a multiple-image experiment and analyzed the obtained diffraction data in the context of the beamline performance. Intensity-error estimations rely on empirical assumptions and are typically underestimated by integration software (Waterman & Evans, 2010 ▶). An objective way to estimate the uncertainty of individual reflections was achieved by investigating the variation of the intensity in the series of diffraction images.

## 2. Experimental

Thaumatin was crystallized by the hanging-drop method using a protein solution of approximately 35 mg ml^−1^ in 50 m*M* HEPES buffer pH 7.0 mixed in a 1:1 ratio with a well solution consisting of 0.75 *M* sodium/potassium tartrate, 0.1 *M* citrate buffer pH 6.5. A tetragonal crystal was grown in space group *P*4_1_2_1_2, with unit-cell parameters *a* = *b* = 57.7, *c* = 149.9 Å. For data acquisition, the crystal was cryoprotected in reservoir solution supplemented with 28%(*v*/*v*) glycerol and cooled in a stream of nitrogen at 100 K delivered by an Oxford Cryostream device.

Diffraction data were collected on beamline 19-BM of the Structural Biology Center at the APS, Argonne National Laboratory (Rosenbaum *et al.*, 2005 ▶) using an ADSC Q210r CCD detector and a wavelength of 0.9792 Å. The APS storage ring operated in the non-top-up mode, with the ring current at about 85 mA at the start of the exposure series. 100 identical images with the same 2° rotation and a 127 mm crystal-to-detector distance were collected successively with the longest crystal axis (*c*) oriented approximately parallel to the detector plane in order to avoid overlap of reflection profiles at the detector window. The beam intensity was not attenuated and an exposure time of 2 s was selected to keep the number of overloaded detector pixels at less than 30 in the first image.

The flux, as measured using a calibrated ion chamber, was 1.01 × 10^11^ photons s^−1^ at the start of the experiment and the beam dimensions at the crystal were 0.051 × 0.075 mm FWHM. The flux density was therefore 2.9 × 10^13^ photons mm^−2^ s^−1^. As estimated by *RADDOSE* (Murray *et al.*, 2004 ▶), the absorbed dose per image was about 0.29 × 10^5^ Gy.

The diffracted intensities were integrated with *DENZO* (Otwinowski & Minor, 1997 ▶) using the ‘oscillation start 0’ command to prevent the program advancing with crystal rotation. The measured intensities (from profile fitting without application of the Lorentz and polarization corrections) in the individual output *.x files of each image were used for further analysis. Only fully recorded and non-overloaded reflections which were present in all 100 images were used in statistical calculations. In order to safely treat each of these reflections as fully recorded, the mosaic spread was overestimated and fixed at 0.5 for each image, whereas the values estimated by *DENZO* were in the range 0.35–0.4. The intensities are presented in analog-to-digital units (ADUs) unless otherwise indicated.

The average intensity of all repeatedly measured reflections in one image decreases with frame number owing to radiation damage and owing to the decay of the current in the storage ring, which operated in non-top-up mode. Over the duration of the experiment the intensities decreased by 7%, while the storage-ring current only diminished by 0.2%. It was therefore decided that it was not necessary to correct the intensities for the decreasing ring current.

The average intensities in the series of frames were fitted with a linear function *y*(*i*) = *I*_0_ + *bi* (where *i* represents the frame number). The intercept *I*_0_ is an estimate of the intensity at the beginning of the experiment, when the crystal has not yet undergone damage, and the slope *b* describes how fast the intensity changes during exposure to X-rays. The same procedure was applied to individual reflections, where some of their intensities increased and some decreased with progressing exposure.

The r.m.s.d. (root-mean-square deviation) of the data points from the linear regression line is calculated by
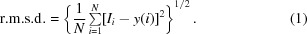

*N* is the number of measurements (here equal to 100), *y*(*i*) is the fitting-function value and *I*_*i*_ is the intensity of a reflection (or the average intensity of all reflections) in frame number *i*. Calculation of the r.m.s.d. therefore represents an alternative method to estimate the uncertainty of individual reflections to that employed by the integration program. In the following, σ_Denzo_ denotes the uncertainty of a reflection estimated by *DENZO* and r.m.s.d. denotes the uncertainty derived from the linear fit describing the variation of the intensity as a function of the frame number.

The relative r.m.s.d. (r.m.s.d._rel_) is calculated by dividing the difference term in the sum by the squared intensity, 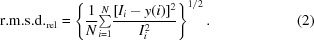



## 3. Results and discussion

### 3.1. Errors in intensity measurement

The measurement of diffraction peak intensities is prone to a variety of experimental errors which consist of random and systematic components. The random part, which affects the precision of the data, arises from effects such as counting statistics associated with the scattering phenomenon itself, sample vibrations caused by cryostream turbulence, flux fluctuations generated by instability of beamline elements, X-ray background noise from noncrystalline material, air or nitrogen scattering, fluctuations in X-ray flux and shutter–spindle synchronization. The systematic components, which affect the accuracy of the data, stem from sample properties, the beamline instruments, the software used for data integration and imperfections in detector calibrations.

In contrast to a standard data-collection protocol, the multi-image experiment minimizes the effects of systematic errors that potentially arise from sample properties and are caused by variations in illuminated crystal volume, absorption and inhomogeneous radiation damage as the irradiated volume is always the same. In terms of the detector, geometric distortions, calibration errors and nonlinear responses are not taken into account, as the same reflections are always measured on the same detector pixels. The error caused by repetitive non-uniformity of the spindle-rotation speed within the narrow oscillation range does not affect the results; however, the remaining spindle range is not probed. The uncertainties evaluated by the multi-image experiment are random in nature and result from the sources listed above.

The error estimation by the integration software can be validated by investigating the variation of the intensity of individual reflections in consecutive images, which is an objective way to estimate the experimental uncertainties.

In summary, the multi-image experiment allows minimization of the effects of systematic errors from the sample and the integration software and allows the influence of the beamline components to be probed.

### 3.2. Effect of radiation damage

**Figure 1:**
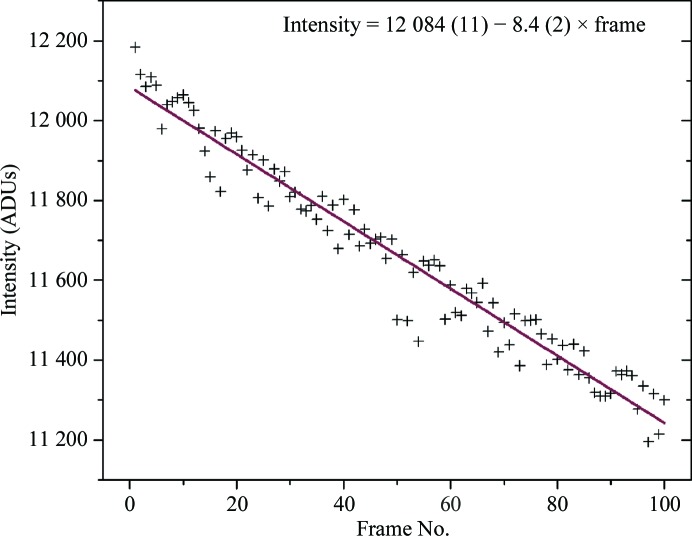
Average intensity of all 4715 fully recorded reflections per diffraction image as a function of frame number. The resolution range is 30–1.4 Å (overloads are excluded). The red line represents the linear fit to the data; the parameters are indicated in the figure.

The behavior of the average intensity of all 4715 measured fully recorded reflections present in all 100 images as a function of frame number is shown in Fig. 1 ▶. The average intensity changes from about 12 100 ADUs in the first image to about 11 200 ADUs in the last image (image 100); that is, by 7%. The decline is monotonic and can be described by a linear function with a corresponding root-mean-square deviation of 53.4. The relative variation of intensities, r.m.s.d._rel_, is very small and amounts to 0.46%, which reflects the high accuracy of the diffraction data and the high stability of the experimental system. If the declining tendency is described by the best least-squares-fitted parabola, the r.m.s.d. value is 50.5. It may be concluded that the linear approximation describes the initial effect of radiation damage well with the modest absorbed dose of 0.29 × 10^5^ Gy per image. Elucidation of the detailed functional character of this effect on the intensities within a wider range of doses would require an increase in exposure or the collection of more images. The total dose of only 2.9 MGy, which is a small fraction of the ‘Garman limit’ of 30 MGy (Owen *et al.*, 2006 ▶) corresponding to the maximum recommended dose, does not permit us to judge whether the exponential model proposed by Blake & Phillips (1962 ▶) and Hendrickson (1976 ▶) describes this effect appropriately, and the linear function was accepted as satisfactory.

**Figure 2:**
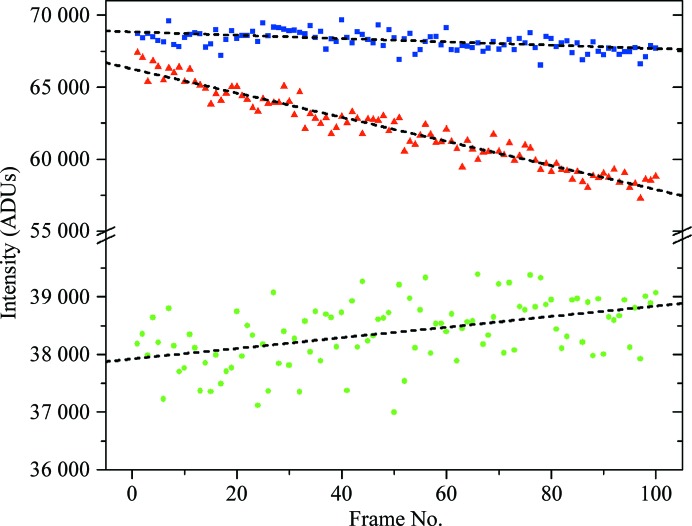
Intensity of three fully recorded reflections as a function of frame number. The break in the grid is from 40 000 to 55 000 ADUs. Blue squares, red triangles and green circles represent the reflections (−12 10 22), (17 −6 −18) and (−15 13 15), respectively. The dotted black lines represent linear regression lines.

Although the average intensity decreases with exposure of the sample to X-rays, individual reflections can behave differently. Fig. 2 ▶ shows the intensity of three reflections as a function of the image number and Table 1 ▶ summarizes the intercept, slope and r.m.s.d. values of the linear regression curves of the analyzed reflections. The intensity of the first reflection (blue squares) decays slowly, similarly to the average intensity of all reflections. The decrease of the second reflection (red triangles), which is initially almost as strong as the first reflection, is more prominent: the intensity drops from 66 000 to 58 000 ADUs and its slope is about eight times larger than that for the first reflection (Table 1 ▶). The third reflection (green spheres) shows a completely different tendency: its intensity increases slightly with absorbed dose. This behavior reflects the structural changes induced by irradiation. Therefore, the decay of a single reflection should not, in most cases, be approximated by the decay of all reflections (Fig. 1 ▶). However, the standard scaling procedures employ one *B* factor per image, implicitly assuming identical deterioration of all reflections during the course of exposure.

### 3.3. Accuracy of the measured intensities

The average values of the intensity, r.m.s.d., r.m.s.d._rel_ and σ_Denzo_ calculated in eight intensity ranges are summarized in Table 2 ▶. The r.m.s.d. values are larger for reflections with high intensities, but their r.m.s.d._rel_, which is normalized to the intensity, is smaller than that of low-intensity reflections. This results from the well known principle of counting statistics that high-intensity reflections, which reflect a larger number of photons, are measured more accurately than those of low intensity. It is interesting to note that the average uncertainty estimated from *DENZO* (σ_Denzo_) is larger than the r.m.s.d. in intensity ranges 1–6, whereas it is smaller in ranges 7 and 8.

A simple model employed by several data-processing programs for the variance σ^2^ of the intensity *I* of a reflection is given by the following equation (Diederichs, 2010 ▶; Evans, 2006 ▶; Leslie, 1999 ▶), 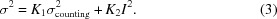

*K*_1_ and *K*_2_ are adjustable parameters. *K*_1_ compensates for errors in gain estimation of CCD detectors by the integration software and partially accounts for a variety of systematic errors, including radiation damage and non-isomorphism. In principle, the gain represents a scale factor between the number of incoming scattered photons and the output detector units (ADUs). The gain is usually approximately estimated from the variation of the background intensity in the pixels around the diffraction peaks, but this procedure does not take into account geometry corrections, flat-field corrections and the point-spread function in CCDs. Another possibility is to use empirical values for the gain, as used for example in *DENZO*, where σ_Denzo_ is evaluated during the integration process by assuming specific default values for each detector type (‘error density’ parameter). Both methods are approximate, which is why it is necessary to use the parameter *K*_1_ to correct the level of uncertainties *a posteriori*. The second term in (3) reflects the systematic components of the instrument-dependent errors, such as those resulting from the detector and beamline elements.

For strong reflections, σ^2^_counting_ can be approximated by the intensity *I*. Rearrangement of (3) then leads to an approximation of the signal-to-noise ratio *I*/σ, 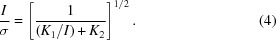
The asymptote of this function is 1/*K*_2_^1/2^; the signal-to-noise ratio *I*/σ is therefore limited and depends on the systematic component of the errors (Diederichs, 2010 ▶). Note that (3) and (4) can be applied to r.m.s.d. or σ_Denzo_ values.

**Figure 3:**
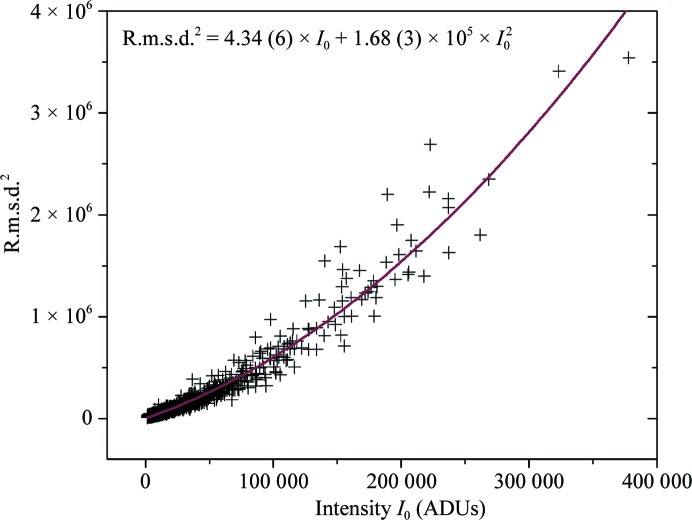
Square of the r.m.s.d. as a function of the extrapolated intensity *I*_0_. The solid line represents the parabolic fit to the data using (3); the fitting parameters are indicated in the plot. The minimum intensity used is 1500 ADUs.

In the following, the experimental uncertainties from the multi-image experiment are compared with those in a recent study by Diederichs (2010 ▶), who analyzed quantitatively the error measurement in a series of data sets from the JCSG archive collected in rotation mode. His study was concerned with the standard diffraction data-collection experiment, whereas our multi-image experiment detects only random errors originating from beamline hardware and minimizes the influence of the sample properties in somewhat idealized experimental conditions. A comparison of numerical values allows an assessment of how certain beamline-dependent factors can change the outcome of the error analysis. For each fully recorded reflection, the square of the r.m.s.d. (variance) is plotted against the extrapolated intensity *I*_0_ in Fig. 3 ▶. For small values of *I*_0_ the growth of r.m.s.d.^2^ is linear, whereas for stronger intensities the *I*_0_^2^ component becomes dominant and r.m.s.d.^2^ increases parabolically. The data can be fitted with a parabolic function using (3), yielding values of 4.34 (6) and 1.68 (3) × 10^−5^ for the parameters *K*_1_ and *K*_2_, respectively. In a study using eight experimental diffraction data sets, *K*_1_ was found to be in the range 4–6 for several different detectors (Diederichs, 2010 ▶). The value of *K*_1_ derived from the multiple-image experiment is therefore in the same range. According to the fitted curve, the value of *K*_2_ amounts to 1.68 (3) × 10^−5^, which is two orders of magnitude smaller than those found in the Diederichs study, where *K*_2_ takes values between 1 × 10^−3^ and 5 × 10^−3^ (note that the parameter *K*_2_ here corresponds to *K*_1_ *K*_2_ in the Diederichs paper). The parameter *K*_2_ is related to the *I*_0_^2^ dependency of the error, a smaller value therefore means that r.m.s.d.^2^ increases more slowly at high intensities and, as a consequence, the asymptotic value of the *I*/r.m.s.d. ratio is larger. For our data, we obtained a value of 244 (as can be calculated using the asymptote of equation 4). This is one order of magnitude higher than the asymptotic value found by Diederichs, which was around 30 for experimental data, and even higher than the value of 161 for a simulated idealized data set.

**Figure 4:**
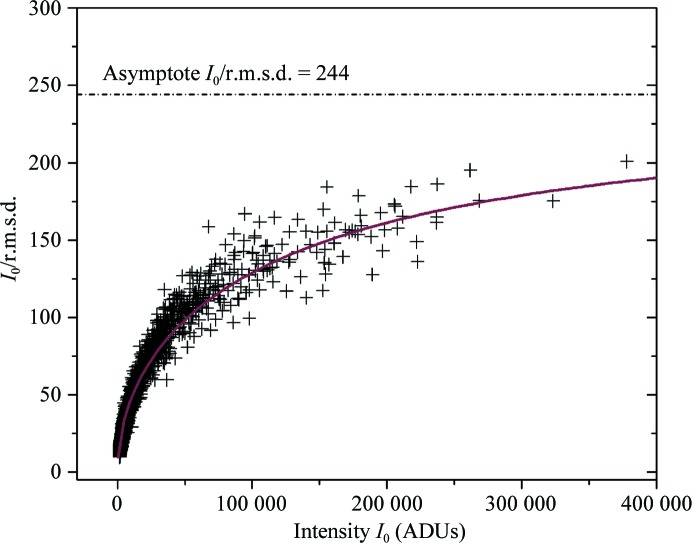
*I*_0_/r.m.s.d. ratio as a function of the intensity *I*_0_. The solid line represents the calculated value of *I*_0_/r.m.s.d. using the fitting parameters from Fig. 3 ▶. The asymptote at *I*_0_/r.m.s.d. = 244 is displayed as a black dotted line.

The *I*_0_/r.m.s.d. ratio as a function of the intensity *I*_0_ is displayed in Fig. 4 ▶. A large part of the data has *I*_0_/r.m.s.d. < 100, but there is a non-negligible number of reflections with even higher signal-to-noise ratios of up to about 170, with the maximum value for the entire data set being 201. Although this ratio is already very high compared with the *I*/σ values reported by Diederichs, it is interesting to note that the data do not reach the asymptotic value of 244. Indeed, intensities of more than one million ADUs would have to be measured in order to reach a level of 90% of the asymptotic value.

The large difference between the values of *K*_2_ found in our study and those derived by Diederichs can be explained by the different experimental setup. Indeed, the present data were obtained in a multiple-image experiment, whereas the previous study was based on conventional data sets which are composed of successive images from a rotating crystal. Besides, *K*_1_ and *K*_2_ were determined for the whole data set from the integration software *XDS* and the sigma values were subsequently calculated using these parameters. On the other hand, our multiple-image experiment allowed us to derive *K*_1_ and *K*_2_ from the r.m.s.d. of the linear regression lines (Fig. 2 ▶).

### 3.4. Contribution of photon statistics to uncertainty

The smallest possible uncertainty of measured diffraction peak intensities is given by the Poisson statistics of the number of photons recorded by the detector. For a non-photon-counting detector, this number has to be established by conversion from the detector output. The best method to determine the conversion factor of the output of the CCD detector (in ADUs) into photon equivalents is to directly record the integrated ADUs of the detector for a known number of photons incident on the face of the detector as follows: an aperture of about the size of a diffraction peak is inserted in front of the detector illuminated by a smooth X-ray field. The flux through the aperture is measured by a photon-counting detector (Bicron) of known quantum efficiency and then recorded by the detector. This avoids the problems of the method discussed above which determines the gain from the statistics of single pixels, which principally leads to incorrect values.

For the ADSC Q210r detector in hardware-binning mode and at a photon energy of 12.66 keV, the conversion factor *c* is *c* = 0.54 photons per ADU as determined by the method described above (Chris Nielsen, ADSC, private communication).

The conversion of the integrated ADUs in a diffraction peak provides the integrated number of incident photons. However, not every incident photon is recorded by the detector. This will reduce the *I*/σ of the signal. A measure of this reduction is the detective quantum efficiency (DQE), which is defined as DQE = [(*I*/σ)_out_/(*I*/σ)_in_]^2^. The highest possible *I*/σ for an incident number *N*_in_ of photons in a diffraction peak is given by assuming Poissonian statistics: (*I*/σ)_in_ = *N*_in_/*N*_in_^1/2^ = *N*_in_^1/2^. For medium to high diffraction peak intensities, the statistics of the photon flux is the dominant contribution to the variance of the CCD detector output. Thus, the DQE is very close to the primary quantum efficiency of the phosphor converting X-rays into visible light flashes. At very low peak intensities, the detector read noise adds significantly to the variance. At very high peak intensities, the analog nature of the CCD limits the increase of *I*/σ, thereby decreasing the DQE. Note that even though a diffraction peak spreads over many pixels with a wide range of ADUs per pixel, the statement above for the DQE of medium to high integrated intensity peaks is still valid since the variances of the pixels with high ADU dominate the total variance, σ^2^_int_ = 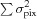 = 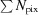 = *N*_int_, and the increased DQE of the pixels with low ADUs has a small effect.

The absorption of the phosphor sheet of the Q210r has been measured to be 0.767 (after scaling from 12.4 to 12.66 keV, which are the photon energy of absorption measurement and the photon energy of this study, respectively). After taking into account the absorption of the phosphor support sheet, the binder and the entrance window, the absorption of the phosphor alone is estimated to be 0.75. This value is used as the DQE of the CCD for the purpose of photon statistics.

The signal-to-error ratio *I*/σ of the recorded diffraction peak intensity is then (*I*/σ)^2^_out_ = DQE × (*I*/σ)^2^_in_ = DQE × *N*_int_, where *N*_int_ is the integrated number of photons of the diffraction peak. Since *N*_int_ = *c* × *I*_int_(ADU), where *I*_int_(ADU) is the integrated number of ADUs of the diffraction peak, (*I*/σ)^2^_out_ = DQE × *c* × *I*_int_(ADU) = 0.75 × 0.54 × *I*_int_(ADU). The signal-to-error ratio *I*/σ of the recorded diffraction peak intensity is then *I*/σ = [0.405 × *I*(ADU)]^1/2^. 

**Figure 5:**
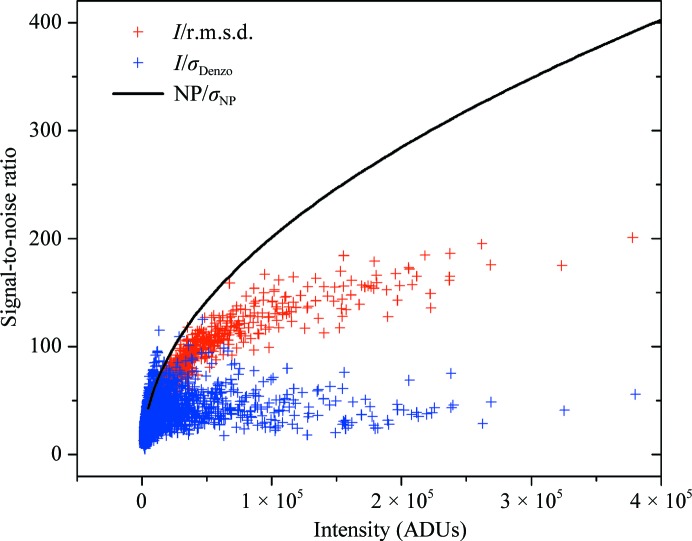
Signal-to-error ratio arising from photon statistics (black line) using the r.m.s.d. (red crosses) and the uncertainty σ_Denzo_ from the *DENZO* integration software (blue crosses).

The signal-to-error ratio owing to photon statistics *I*/σ is plotted against the intensity (in ADU) in Fig. 5 ▶. The level of intensity where this curve markedly differs from the *I*/r.m.s.d. or *I*/σ_Denzo_ of the measured intensities indicates where other factors, such as beamline instruments, crystal properties or detector properties other than the quantum efficiency of the phosphor, become dominant in the uncertainty of the diffraction data.

For weaker intensities (<30 000 ADUs), the *I*/σ_Denzo_ ratio follows Poissonian statistics and reaches values of up to 100. However, the signal-to-noise ratio does not increase further for high intensities. On the other hand, the *I*/r.m.s.d. values follow the Poissonian curve for weaker reflections of <50 000 ADUs and continue to grow more slowly afterwards. The uncertainties derived by our method are therefore less affected by systematic errors induced by the beamline elements, the detector or the crystal itself.

### 3.5. Analysis of uncertainties derived from *DENZO*

**Figure 6:**
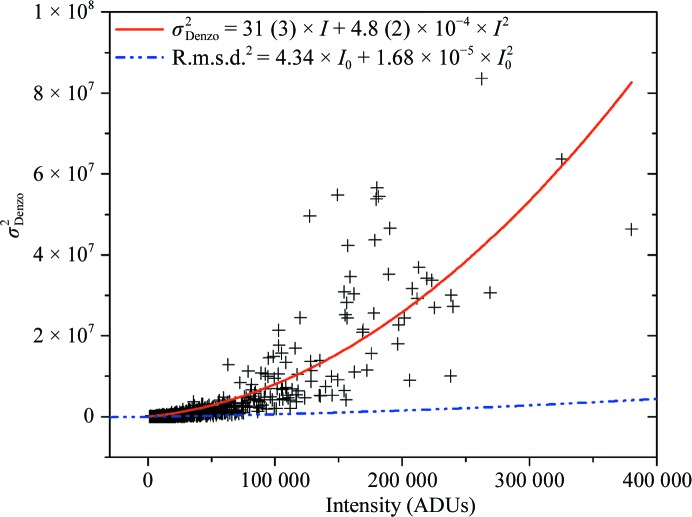
Square of σ_Denzo_ as a function of the corresponding measured intensity *I* taken from the first frame of the multi-image experiment. The solid line corresponds to the parabolic fit (using equation 3) and the dotted line represents the fit line shown in Fig. 3 ▶ using the squared r.m.s.d. values as a function of the extrapolated intensity *I*_0_. The minimum intensity used was 1500 ADUs.

Fig. 6 ▶ displays the squared σ_Denzo_ as a function of the measured intensity *I*. The data seem to have a parabolic distribution and can be fitted using (3). However, the σ^2^_Denzo_ values at lower intensities, between 30 000 and 70 000, are not well represented by the resulting fitting curve. This might partially be a consequence of the behavior of σ^2^_Denzo_ at high intensities. Using only intensities of <150 000 for the fit of the parabola (data not shown) a negative value of *K*_1_ is obtained, whereas a positive value is expected because *K*_1_ is related to the error in gain. Thus, the behavior of σ^2^_Denzo_ does not agree with the expected parabolic function.

For comparison, the fit derived from r.m.s.d.^2^ as in Fig. 3 ▶ is also displayed (blue dotted line). Clearly, σ^2^_Denzo_ increases much more rapidly than r.m.s.d.^2^, as had previously been indicated by comparing the average values of 〈σ_Denzo_〉 and 〈r.m.s.d.〉 in intensity ranges (Table 2 ▶). The asymptote of *I*/σ_Denzo_, as derived from the parameter *K*_2_, amounts to 46, which is significantly smaller than the value of 244 derived from the r.m.s.d. Indeed, the estimation of the σ_Denzo_ values is roughly optimized for ‘classic’ rotational data-collection strategies using the empirical *DENZO* ‘error density’ (gain) parameter, where multiple error sources are present such as imperfect beam centering, varying irradiated volume resulting in non-uniform radiation damage and absorption, and where only a restricted number of redundant reflections is available. It is therefore likely that the σ_Denzo_ errors are overestimated in the case of our multiple-image experiment, which had been designed to avoid some of these error elements. Hence, it is preferable to use the r.m.s.d. values in the context of assessing the performance of a beamline, as they reflect the variation in intensity of repeatedly measured identical reflections.

## 4. Conclusions

A multi-image experiment based on repeatedly measuring analogous diffraction images at the same crystal orientation was performed. The analysis of 100 consecutive diffraction frames allowed us to experimentally derive the uncertainties of the measured intensities by calculating the root-mean-square deviation from the observed variability of intensities in consecutive images. It was shown that the asymptote of the *I*/r.m.s.d. curve is much higher than that of the signal-to-noise ratio (*I*/σ) determined from complete data sets in rotation mode. The multi-image experiment minimizes the influence of crystal properties on the quality of the diffraction data. We conclude that the multiple-image experiment is a simple and adequate method to learn about some important aspects of beamline components.

## Figures and Tables

**Table 1:** Intercept, slope, r.m.s.d. and r.m.s.d._rel_ for the curves in Figs. 1 ▶ and 2 ▶

Reflection	Intercept	Slope	R.m.s.d.	R.m.s.d._rel_ (%)
All	12084	−8.4	53.4	0.46
−12 10 22	68851	−11.7	538	0.79
17 −6 −18	66277	−83.9	626	1.01
−15 13 15	37922	9.2	487	1.28

**Table 2:** Average values of r.m.s.d., r.m.s.d._rel_, intensity 〈*I*〉 and 〈σ_Denzo_〉 calculated in eight intensity ranges The number of reflections and the lower intensity limit per bin are indicated in the second and third columns, respectively. 〈σ_Denzo_〉 and 〈*I*〉 are calculated for the first image; the r.m.s.d. is calculated on the basis of 100 images.

Bin	No.	Intensity range (ADUs)	R.m.s.d.	R.m.s.d._rel_ (%)	〈σ_Denzo_〉	〈*I*〉
1	110	75000	938	0.74	3594	136428
2	81	50000	547	0.93	1365	61397
3	93	35000	428	1.10	978	41207
4	201	20000	341	1.34	656	26986
5	314	10000	250	1.92	354	14141
6	449	5000	192	2.92	215	7266
7	524	2500	151	4.69	127	3586
8	446	1500	127	7.27	93	1946
